# Cathepsin-L Can Resist Lysis by Human Serum in *Trypanosoma brucei brucei*

**DOI:** 10.1371/journal.ppat.1004130

**Published:** 2014-05-15

**Authors:** Sam Alsford, Rachel B. Currier, José Afonso Guerra-Assunção, Taane G. Clark, David Horn

**Affiliations:** 1 London School of Hygiene & Tropical Medicine, London, United Kingdom; 2 Division of Biological Chemistry & Drug Discovery, College of Life Sciences, University of Dundee, Dundee, United Kingdom; Washington University School of Medicine, United States of America

## Abstract

Closely related African trypanosomes cause lethal diseases but display distinct host ranges. Specifically, *Trypanosoma brucei brucei* causes nagana in livestock but fails to infect humans, while *Trypanosoma brucei gambiense* and *Trypanosoma brucei rhodesiense* cause sleeping sickness in humans. *T. b. brucei* fails to infect humans because it is sensitive to innate immune complexes found in normal human serum known as trypanolytic factor (TLF) 1 and 2; the lytic component is apolipoprotein-L1 in both TLFs. TLF resistance mechanisms of *T. b. gambiense* and *T. b. rhodesiense* are now known to arise through either gain or loss-of-function, but our understanding of factors that render *T. b. brucei* susceptible to lysis by human serum remains incomplete. We conducted a genome-scale RNA interference (RNAi) library screen for reduced sensitivity to human serum. Among only four high-confidence ‘hits’ were all three genes previously shown to sensitize *T. b. brucei* to human serum, the haptoglobin-haemoglobin receptor (HpHbR), inhibitor of cysteine peptidase (ICP) and the lysosomal protein, p67, thereby demonstrating the pivotal roles these factors play. The fourth gene identified encodes a predicted protein with eleven *trans*-membrane domains. Using chemical and genetic approaches, we show that ICP sensitizes *T. b. brucei* to human serum by modulating the essential cathepsin, CATL, a lysosomal cysteine peptidase. A second cathepsin, CATB, likely to be dispensable for growth in *in vitro* culture, has little or no impact on human-serum sensitivity. Our findings reveal major and novel determinants of human-serum sensitivity in *T. b. brucei*. They also shed light on the lysosomal protein-protein interactions that render *T. b. brucei* exquisitely sensitive to lytic factors in human serum, and indicate that CATL, an important potential drug target, has the capacity to resist these factors.

## Author Summary

The interplay among host innate immunity and resistance mechanisms in African trypanosomes has a major impact on the host range of these tsetse-fly transmitted parasites, defining their ability to cause disease in humans. A genome-scale RNAi screen identified a highly restricted set of four genes that sensitise trypanosomes to human serum: those encoding the haptoglobin-haemoglobin receptor, a predicted *trans*-membrane channel, a lysosomal membrane-protein and the cysteine peptidase inhibitor. An analysis of the cysteine peptidases revealed cathepsin-L as the protease regulated by the inhibitor – and with the capacity to render the parasite resistant to lysis by human serum. These findings emphasise the importance of parasite factors for the delivery and stability of host toxins. They also shed light on the control of proteolysis by parasites and potential unanticipated consequences of therapies that target the parasite proteases.

## Introduction

The African trypanosomes are flagellated protozoan parasites comprising several species of the genus *Trypanosoma*, which cause devastating diseases in humans and livestock. One key feature that distinguishes members of this group is their sensitivity to innate trypanolytic factors (TLFs) found in human serum. *T. b. brucei* and related species cause nagana in livestock but these parasites are rapidly lysed by human TLFs [1], [2]. *T. b. gambiense* and *T. b. rhodesiense*, on the other hand, although sharing >99% genome sequence identity with *T. b. brucei* [3], have evolved distinct mechanisms to escape lysis by human serum; these are the causative agents of human African trypanosomiasis (HAT), also known as sleeping sickness, in Western and Eastern Africa, respectively. *T. b. gambiense* is responsible for 97% of reported cases of HAT [4].

There are two classes of TLF found in normal human serum, TLF-1, which is a component of high density lipoprotein [5], [6], and TLF-2, which is an apolipoprotein-A1/IgM complex [7], [8]; the active lytic component in both TLFs is apolipoprotein-L1 (APOL1) [9]. Both TLFs also contain haptoglobin-related protein, which, in the case of TLF-1, mediates binding to the *T. b. brucei* haptoglobin-haemoglobin receptor (HpHbR) and uptake into the cell [10], [11]. Following uptake, APOL1 is inserted into endosomal and lysosomal membranes, where Bcl-2-like pore-formation is thought to be responsible for osmotic swelling and lysis [12], [13].

Human TLF resistance mechanisms of *T. b. gambiense* and *T. b rhodesiense* have now been described, and these involve reduced TLF binding/uptake, APOL1 sequestration, or reduced APOL1 toxicity, possibly due to membrane stiffening. Reduced TLF binding/uptake operates in *T. b. gambiense* due to reduced expression of HpHbR and/or mutations in HpHbR [14]–[16]. Endosomal sequestration of APOL1 operates in *T. b. rhodesiense* due to the expression of a serum resistance-associated protein (SRA) related to a glycosyl-phoshatidylinositol membrane-anchored variant surface glycoprotein (VSG) [2], [17]. Expression of a VSG-related protein also confers TLF-resistance to *T. b. gambiense* [18], [19], but in this case the VSG-like *T. b. gambiense*-specific glycoprotein or TgsGP may protect cells from APOL1 by stiffening endosomal membranes rather than through direct interaction with, or sequestration of, APOL1 [19].

The lysosomal membrane protein, p67 [20] and inhibitor of cysteine peptidase (ICP) [19] have also been shown to contribute to human TLF susceptibility using loss-of-function approaches in *T. b. brucei*. While HpHbR plays a role in TLF binding/uptake, the mechanism by which p67 contributes to human serum sensitivity in *T. b. brucei* remains unknown. Depletion of p67 causes lysosomal dysfunction, but does not increase lysosomal pH [20]; acidification has been proposed to be important for the insertion of APOL1 into membranes and the resulting lytic activity [12], [13], [21]. The role of the individual cysteine peptidases, the targets of ICP, has not previously been investigated, although *T. b. brucei* and *T. b. gambiense* cells exposed to a cysteine peptidase inhibitor display increased accumulation of TLF-1 [2] and APOL1 [19], strongly suggesting that a cysteine peptidase contributes to the destruction of APOL1. Cysteine peptidase inhibition by ICP likely similarly increases APOLI accumulation, explaining increased human serum resistance following ICP knockdown [19]. Thus, gain-of-function, through the expression of modified VSGs, or loss of TLF-receptor function, have contributed to the emergence of human-infective African trypanosomes. However, other undiscovered resistance mechanisms are thought to operate in these parasites [22]; expression of TgsGP does not confer human serum resistance to *T. b. brucei* [23], and the main route of entry for TLF-2 in *T. b. brucei* is thought to be independent of HpHbR [10], [19].

We sought to confirm those factors known to render *T. b. brucei* susceptible to lysis by human serum and to screen for additional factors. A genome-scale RNA interference library screen for increased resistance to human serum identified all three known genes and only one additional gene, encoding a novel putative *trans*-membrane channel, with high-confidence. This library was previously shown to yield read-outs representing approximately 5-fold genome coverage, or more than 99% of the >7,000 non-redundant protein coding sequences in the *T. b. brucei* genome [24], and an approach related to the one described here was used to identify efficacy determinants for all five current anti-HAT drugs [25]. We next explored the unexplained role of the cysteine peptidase inhibitor in this process, and show that ICP impacts human serum resistance by specifically modulating the activity of the lysosomal cysteine peptidase, cathepsin-L (CATL).

## Results

### A genome-scale screen for genes controlling human serum sensitivity in *T. b. brucei*

**Figure 1:**
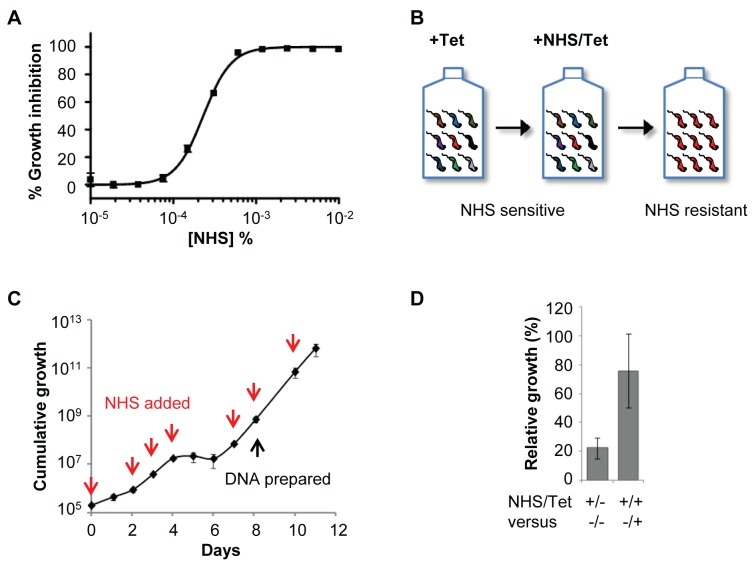
A genome-scale *T. b. brucei* RNAi library screening strategy for human serum resistance. (**A**) *T. b. brucei* (MITat 1.2; 2T1) have an EC_50_ of less than 0.00025% normal human serum (NHS) when incubated for 72 hours at 37°C. (**B**) Schematic showing selection of NHS-resistant parasites from the RNAi library. (**C**) A population resistant to NHS was selected. RNAi was induced in 1 µg/ml tetracycline (Tet) for 24 hours prior to selection initiated at day 0; red arrows indicate culture dilution and addition of fresh NHS and tetracycline at 0.0005% and 1 µg/ml, respectively. (**D**) The NHS resistance phenotype of the selected library was tetracycline-inducible; error bars represent standard deviation.

Natural hosts for bloodstream form (BSF) *T. b. brucei* include bovids, and these parasites are typically propagated in a culture medium containing 10% bovine serum. In this culture environment, the half maximal effective growth-inhibitory concentration (EC_50_) of normal human serum (NHS) against cultured BSF *T. b. brucei* was less than 0.00025% (Figure 1A), revealing the exquisite sensitivity of these parasites to lytic factors in NHS. To identify *T. b. brucei* factors that contribute to the trypanolytic activity of NHS, we selected a multi-genome coverage BSF *T. b. brucei* RNAi library in 0.0005% NHS (see Figure 1B). Using this loss-of-function approach, knockdown of factors that normally contribute to human serum sensitivity will generate cells with increased resistance to this toxin. Under RNAi-inducing conditions, population growth was severely curtailed for six days in the presence of NHS; the human serum was added to the growth medium 24 h after inducing RNAi with tetracycline (Figure 1C). A population that displayed tetracycline-dependent tolerance of this concentration of NHS emerged thereafter (Figure 1D), and was harvested for DNA extraction and RNA interference target sequencing (RIT-seq) two days later.

**Figure 2:**
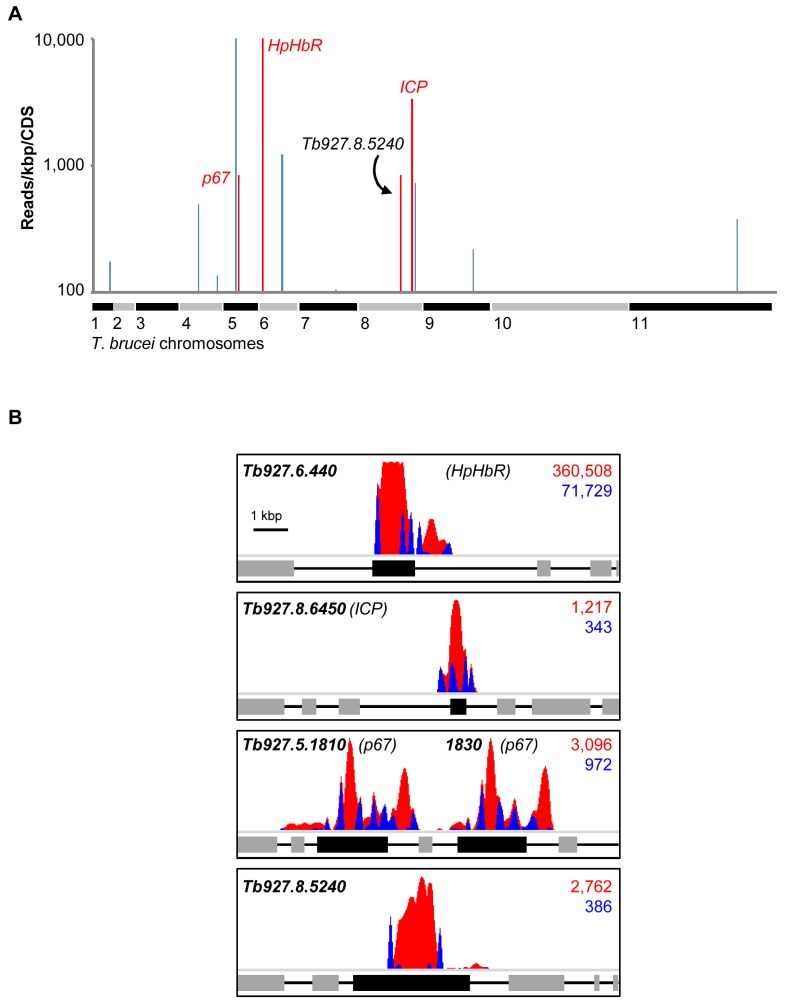
An RNAi screen reveals major determinants of human serum sensitivity in *T. b. brucei*. (**A**) Genome-wide NHS RIT-seq profile representing 7,433 non-redundant protein-coding sequences, showing those represented by more than 100 reads per kbp; three known human serum sensitivity determinants, *HpHbR* (Tb927.6.440), *p67* (Tb927.5.1810/1830) and *ICP* (Tb927.8.6450), and the only other high-confidence and novel ‘hit’ (Tb927.8.5240) are highlighted in red. (**B**) Profiles of mapped RIT-seq reads for the four high-confidence hits identified in (A); boxes represent protein coding sequences; total reads (red) and tagged reads (blue; containing the 14-bp RNAi construct signature sequence) mapped to each region are shown in the top right corner of each panel; see Table 1 for further details.

Using a modified RIT-seq [24] methodology (see Materials and Methods), we generated and mapped individual sequence reads representing the human serum-enriched RNAi target fragments, about 0.5 million reads in total. Approximately 24% of these reads incorporated a 14-bp RNAi construct signature found at the junction with each gene-specific RNAi target fragment, and this allowed us to focus on only ‘high-confidence hits’: genes identified in the screen by more than 99 reads per kilobase per CDS (Figure 2A), with more than 99 reads containing the RNAi construct signature, and at least two independent RNAi target fragments (Table 1). We previously applied similarly stringent criteria to define the key efficacy determinants of the anti-HAT drugs [25].

**Table 1:** Summary of RIT-seq outputs following RNAi library selection in NHS.

		Reads			RNAi	Fitness
Gene ID	Annotation^a^	Total	‘Tagged’^b^	Total/kbp^c^	targets/CDS^d^	ratio^e^
**High-confidence hits**						
Tb927.6.440	HpHbR	360,508	71,729	297,449	3	0.14
Tb927.8.6450	ICP	1,217	343	3,325	2	2
Tb927.5.1810	p67	1,648	531	832	4	0
Tb927.5.1830	p67	1,448	441	729	3	0.31
Tb927.8.5240	Conserved hypothetical	2,762	386	833	2	1.38
**Other hits** f						
Tb927.5.1540	Conserved hypothetical	100,080	35,814	45,326	**1**	1.24
Tb927.6.2930	Conserved hypothetical	1,930	241	1,214	**1**	0.41
Tb927.8.6870	Conserved hypothetical	1,805	409	719	4	32.6
Tb927.4.2090	Hypothetical	252	**94**	488	**1**	0.68
Tb927.11.12880	Conserved hypothetical	161	**88**	378	**1**	2.11
Tb927.9.11000	VSG pseudogene	90	**34**	214	**1**	0.53
Tb927.1.3730	Hypothetical	30	**15**	172	**1**	0.08
Tb927.4.4350	Hypothetical	82	**41**	134	**1**	0.81
Tb927.7.4500	Hypothetical	166	**55**	105	3	0.83
Tb927.8.2380	ABC transporter	350	**64**	101	3	0.89

a Annotations derived from GeneDB, http://www.genedb.org/Homepage/Tbruceibrucei927.

b ‘Tagged’ reads incorporate a 14-bp RNAi construct signature.

c Plotted in Figure 2A.

d High-confidence ‘hits’ characterised by >99 reads containing the 14-base RNAi construct signature, and >1 distinct RNAi target fragment.

e Bloodstream-form (BSF) *T. b. brucei* RNAi library fitness ratio derived from reference [24]; six-day induced read count divided by uninduced read count.

f Other ‘hits’ are characterised by massive gain-of-fitness during RNAi induction (Tb927.8.6870) [24], a single RNAi target sequence (Tb927.5.1540, Tb927.6.2930), or <100 reads containing the 14-bp RNAi construct signature (relevant criteria highlighted in bold).

**Figure 3:**
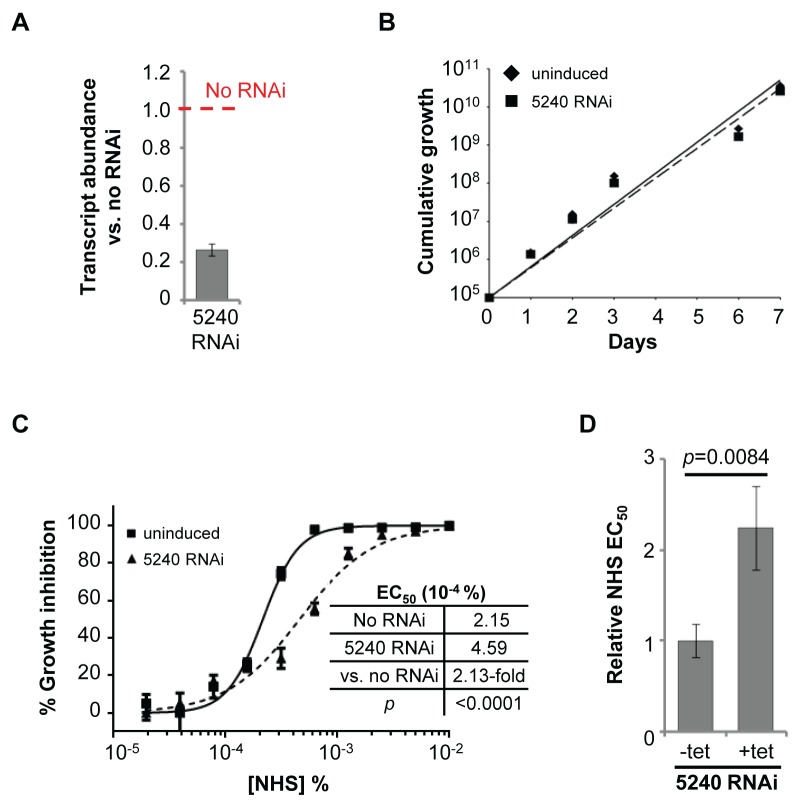
Specific RNAi-mediated depletion of Tb927.8.5240 renders cells less sensitive to NHS. (**A**) Tb927.8.5240 depletion, as demonstrated by reverse transcriptase qPCR (see also Tables S1 and S2 in Text S1), has no significant effect on parasite population growth (**B**); bars represent standard deviation from three independent strains. (**C**, **D**) RNAi against Tb927.8.5240 increases NHS EC_50_; bars represent standard error (C) or standard deviation (D); *p* values derived from extra sum-of-squares F-test (C) or paired t-test (D).

As detailed above, three *T. b. brucei* genes have been shown to play a role in trypanolysis by NHS. Remarkably, we identified only four high-confidence hits in our screen. The presence of the three known genes within this set (Figure 2A), haptoglobin-haemoglobin receptor (HpHbR) [11], inhibitor of cysteine peptidase (ICP) [19], and the lysosomal membrane protein, p67 [20], provides excellent validation for the RNAi-screening approach. The schematic in Figure 2B shows the four high-confidence loci identified in the screen with mapped sequence reads. The novel high-confidence hit (Tb927.8.5240) encodes a ‘conserved hypothetical’ protein (Figure 2A and Table 1). Although it is likely membrane-associated, as it contains 11 putative *trans*-membrane domains, we have been unable to establish its sub-cellular localisation by *C*-terminal epitope tagging (data not shown). Specific stem-loop RNAi depletion of Tb927.8.5240 in three independent cell lines, confirmed by quantitative reverse transcriptase PCR (Figure 3A; Text S1), had no significant effect on bloodstream-form population growth over seven days (Figure 3B), but resulted in a 2.3-fold average increase in NHS EC_50_, demonstrating its contribution to NHS-sensitivity (Figure 3C, D).

The additional genes highlighted in blue in Figure 2A are listed in Table 1. These failed to fulfil the stringent criteria for further analysis detailed above. The only exception being Tb927.8.6870 whose knockdown has previously been shown to lead to a significant gain of fitness [24]; we subsequently confirmed that loss of this protein did not influence sensitivity to NHS (data not shown).

### Cathepsins fail to resist human serum trypanolytic activity in the presence of ICP

ICPs are conserved in protozoal and bacterial pathogens [26]. The *T. b. brucei* and *T. b. gambiense ICP* genes are almost identical and are predicted to encode proteins of 13.5 kDa. They are thought to block cysteine peptidase activity by occupying the substrate-binding cleft [27], and to play a role in regulating parasite infectivity and VSG coat exchange during differentiation [28]. African trypanosomes express two cathepsins, CATB and CATL [29], which are highly conserved between *T. b. brucei* and *T. b. gambiense*, and at least one (CATL) localises to the lysosome [30]. We used chemical and genetic approaches to explore the potential roles of *T. b. brucei* CATB and CATL in resisting lysis by human serum.

**Figure 4:**
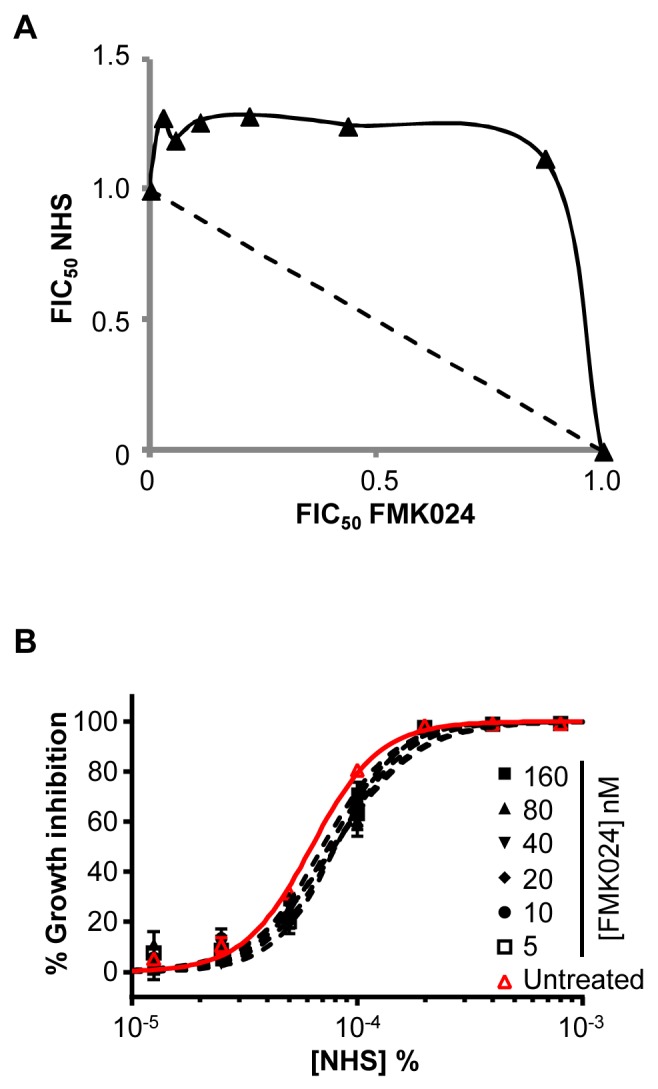
Chemical inhibition of *T. b. brucei* cathepsins in the presence of ICP has no effect on human serum trypanolytic activity. (**A**) FMK024 fails to synergise with NHS against wild-type *T. b. brucei*; isobologram analysis showing 50% fractional inhibitory concentrations (FIC); dashed line indicates the expected output for no interaction. (**B**) Individual NHS EC_50_ curves generated in the presence of 5 to 160 nM FMK024; bars represent standard error.

Initially, we tested the dual CATB/L inhibitor, FMK024, in combination with NHS against *T. b. brucei*. Isobologram and EC_50_ analyses revealed that this inhibitor fails to synergise with NHS in cell-killing assays (Figure 4A, B). Indeed, the addition of increasing amounts of FMK024 causes little change in parasite sensitivity to NHS (Figure 4B), suggesting that the inhibitory function of endogenous ICP may be modulated as a consequence of changes in protease activity elicited by exogenous inhibitor. FMK024 applied at 10 or 20 µM (62.5 and 125-fold higher than the highest concentration used here) has been shown to cause lysosomal accumulation of TLF in *T. b. gambiense* [19] and of APOL1 in SRA-expressing *T. b. brucei* [2], respectively. It should be noted, however, that such high concentrations of FMK024 would likely lead to total inhibition of lysosomal cathepsin activity, and it is unlikely that ICP modulation would have any impact. Indeed, FMK024 treatment is lethal at these concentrations in our EC_50_ assays, independent of NHS exposure (see below).

**Figure 5:**
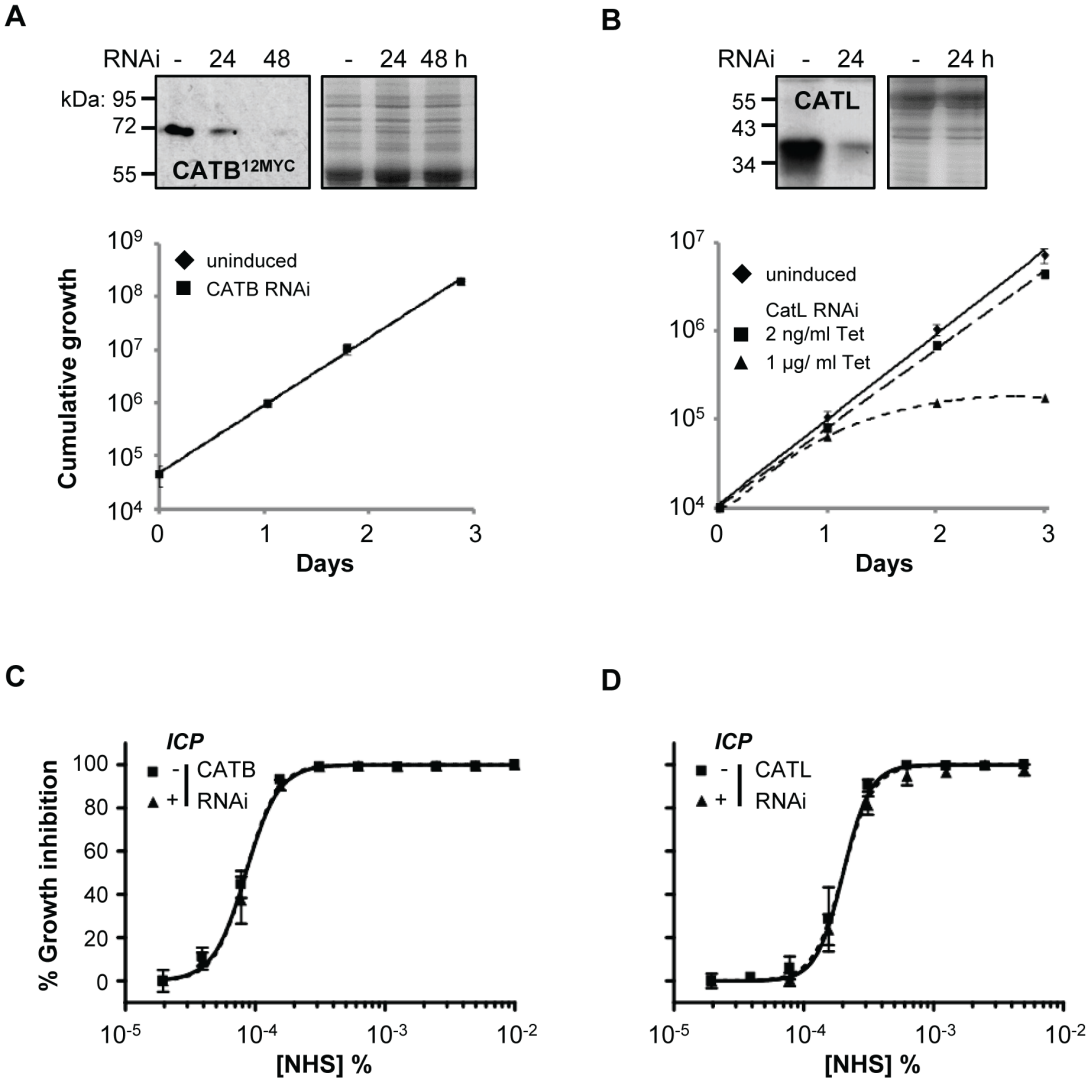
Specific depletion of *T. b. brucei* cathepsins in the presence of ICP has no effect on human serum trypanolytic activity. (**A**) CATB depletion has no effect on cell growth, while (**B**) induction of CATL RNAi in 1 µg/ml tetracycline (Tet) results in a significant growth defect; bars represent standard deviation from four and two independent strains, respectively. CATB^12MYC^ and anti-CATL western blots confirm depletion of the individual cathepsins; coomassie-stained gels show loading. Representative NHS EC_50_ analysis following (**C**) CATB and (**D**) CATL depletion in wild-type *T. b. brucei*; RNAi induced in 1 µg/ml and 2 ng/ml tetracycline, respectively; bars represent standard error.

We next used RNAi to knockdown CATB or CATL individually in *T. b. brucei*. Specific protein depletion was confirmed by western blot, and subsequent analyses revealed that only CATL activity appears to be particularly important for robust growth (Figure 5A, B). Previous findings suggested that CATB but not CATL was essential for growth [31], [32]; however, our results are consistent with the recent chemical and genetic validation of CATL as a more appropriate drug target [25], [29]. Although we used a sub-lethal knockdown in the case of CATL, we were able to obtain substantial protein depletion compatible with continued growth [25] (Figure 5B). Consistent with the results obtained above using chemical inhibition of cathepsin activity, both knockdowns failed to synergise with NHS in killing *T. b. brucei* (Figure 5C, D). These data suggest that if a protease can resist lysis by human serum, its activity is suppressed.

### CATL resists human serum trypanolytic activity in the absence of ICP

**Figure 6:**
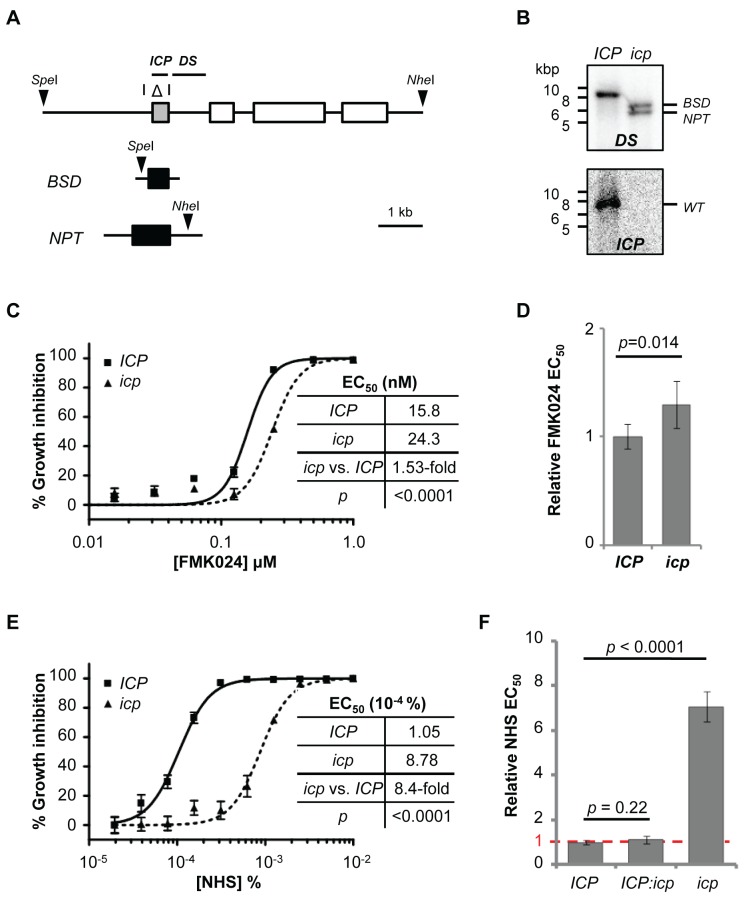
Disruption of *ICP* in *T. b. brucei* renders cells less sensitive to NHS. (**A**) Schematic showing segment deleted from the *ICP* locus (highlighted with an open triangle), and positions of restriction sites (arrow heads), and *ICP* and downstream (DS) flanking probes. (**B**) Southern blots confirming *ICP* deletion from the 2T1 cell line; *BSD*, blasticidin-S-deaminase; *NPT*, neomycin phosphotransferase. (**C**, **D**) ICP loss has a minor but significant effect on FMK024 sensitivity (mean fold EC_50_ change, 1.31; five independent strains). (**E**) NHS EC_50_ analysis of a representative *icp* null strain; bars represent standard error. (**F**) Reduced NHS sensitivity requires deletion of both *ICP* alleles (five independent strains); loss of one *ICP* allele has no significant effect on sensitivity to NHS (two independent strains). Bars represent standard error (C, E) and standard deviation (D, F); *p* values derived from extra sum-of-squares F-test (C, E) or paired t-test (D, F).

The results above show either that repression of cathepsin activity by ICP renders *T. b. brucei* sensitive to human serum and that cathepsin knockdown is compensated for by down-regulation of ICP activity, or that CATB and CATL play no role in resistance to lysis by human serum. To distinguish between these possibilities, we generated *icp* null *T. b. brucei* [33] (Figure 6A, B). As previously shown for a distinct cathepsin inhibitor [28], the *icp* null strains displayed a minor but significant increase in FMK024 EC_50_ (Figure 6C, D), confirming up-regulation of a cathepsin activity required for robust growth, most-likely that due to the essential CATL (see above). The *icp* null strains were, on average, 7.2-fold less sensitive to NHS (Figure 6E, F), validating this RNAi screening output and also consistent with a recent report [19].

**Figure 7:**
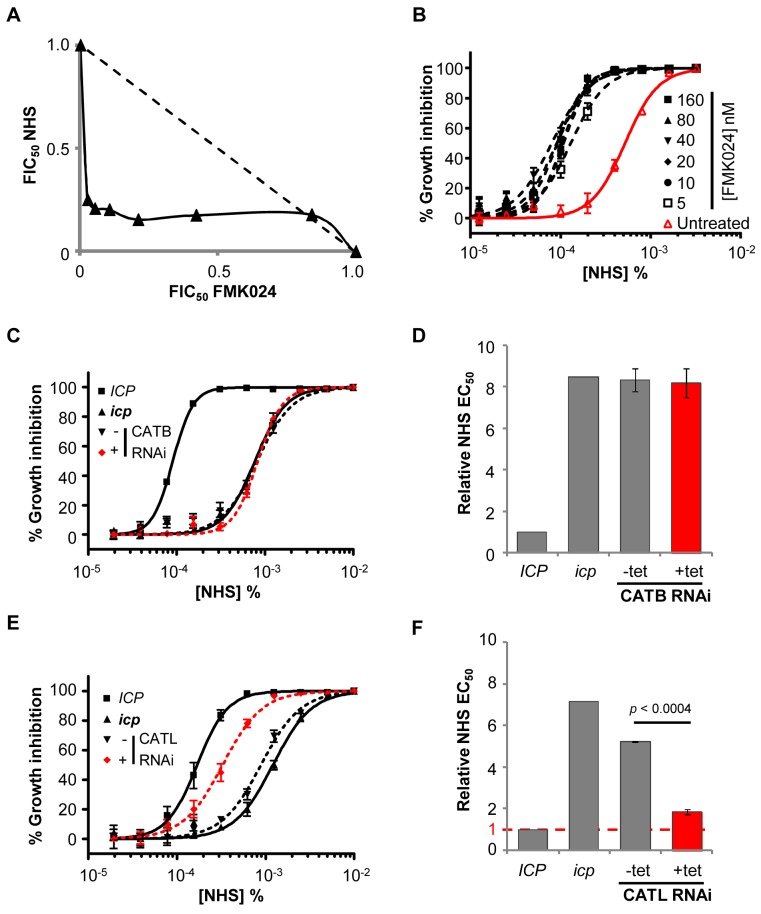
CATL resists human serum trypanolytic activity in the absence of ICP. (**A**) FMK024 and NHS act synergistically against *icp* null *T. b. brucei*; isobologram analysis showing 50% fractional inhibitory concentrations (FIC) where the dashed line indicates the expected output for no interaction. (**B**) Treatment with 5–160 nM FMK024 reverses the *icp* null *T. b. brucei* NHS resistance phenotype. (**C**) Representative NHS EC_50_ analysis following CATB depletion in *icp* null compared with wild-type and *icp* null *T. b. brucei* parental cell lines. (**D**) NHS EC_50_ analysis following CATB depletion in four independent strains. (**E**) Representative EC_50_ analysis following CATL depletion in *icp* null cells compared with wild-type and *icp* null *T. b. brucei* parental cells. (**F**) NHS EC_50_ analysis of two independent CATL RNAi *icp* null strains. Bars represent standard error (C, E) and standard deviation (D, F); *p* value derived from paired t-test; CATB and CATL knockdowns were induced in 1 µg or 2 ng/ml tetracycline, respectively.

In striking contrast to the situation in wild-type *T. b. brucei*, isobologram and EC_50_ analyses revealed strong synergy between FMK024 and NHS in killing *icp* null *T. b. brucei* (Figure 7A). Indeed, in the presence of 5 to 160 nM FMK024, the NHS sensitivity was almost completely reversed to that of wild-type cells (Figure 7B). These results suggest that one or both of the cathepsins can indeed confer resistance to lysis by human serum, but only effectively in the absence of ICP. We next set out to determine which of the cathepsins is responsible for this phenotype. In order to assess the contribution of the individual cathepsins to resisting NHS, we generated strains for the inducible RNAi-mediated knockdown of either CATB or CATL in an *icp* null background. Once again, we had to use a sub-lethal knockdown in the case of CATL (see Figure 5B).

CATB knockdown in these strains had no impact on NHS-sensitivity (Figure 7C, D). Hence, although CATB may have a role in the degradation of other host-derived proteins, including transferrin [32], our data suggests that it does not target human serum lytic factors. In contrast, CATL knockdown was associated with a highly significant increase in sensitivity to NHS (Figure 7E, F). Failure to completely reverse the NHS-resistance phenotype following CATL RNAi, may be explained by a second contributing factor or, more likely in our view, is because these experiments had to be carried out under partial knockdown conditions. We conclude that ICP increases sensitivity to NHS primarily by inhibiting CATL activity.

## Discussion

*T. b. gambiense* and *T. b. rhodesiense* can resist the APOL1-based trypanolytic factors found in normal human serum, while *T. b. brucei* fails to do so. We report here an RNAi library screen in bloodstream-form *T. b. brucei* for resistance to human serum and identify all three known genes, as well as a novel gene, that increase *T. b. brucei* susceptibility to this innate immune defence mechanism. We go on to show that one of these genes, encoding inhibitor of cysteine peptidase, acts by modulating the essential activity of CATL, a lysosomal cysteine peptidase. These findings illuminate the interactions between ICP, CATL and human serum, and have important implications for human infectivity, as well as for therapies based on cathepsin inhibitors [34] or serum lytic factors [35]. Finally, we have revealed a novel role for a putative *trans*-membrane domain protein, Tb927.8.5240, in determining sensitivity to NHS.

A loss-of-function phenotype, associated with HpHbR [14]–[16], contributes to human serum resistance in *T. b. gambiense*, the most prevalent cause of sleeping sickness. As expected, our RNAi library screen for human serum resistance identified the gene encoding this protein and also the gene encoding the lysosomal membrane protein, p67, also previously linked to this phenotype through experimental loss-of-function analysis [20]. This confirmed the power and utility of the RNAi-screening approach. Our screen also identified the gene encoding ICP, which was recently linked to human serum sensitivity by others [19], and a fourth, novel gene (Tb927.8.5240), encoding a predicted multi-pass *trans*-membrane protein, with an almost identical homolog in *T. b. gambiense*. These outputs indicate a remarkably low rate of false positives, and suggest a similarly low rate of false negatives when using a multi-genome coverage RNAi library to identify high-confidence hits in *T. b. brucei* (see Materials and Methods).

**Figure 8:**
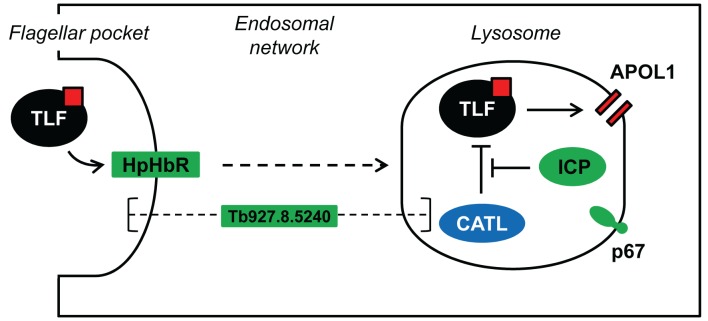
Model showing the proposed interactions among ICP, CATL and TLF. The four proteins identified in our loss-of-function RNAi library screen are highlighted in green. TLF, including APOL1 (red square), is taken up via HpHbR in the parasite's flagellar pocket, the primary site of endocytosis in *T. b. brucei*. The lytic factor transits the endosomal system, eventually reaching the lysosome. APOL1 is thought to form pores in the lysosomal membrane, ultimately leading to cellular lysis. CATL activity is normally tightly regulated by ICP; however, we speculate that loss of ICP increases resistance to human serum due to increased proteolysis of TLF, and possibly APOL1, by CATL. The sub-cellular localisation of the putative channel protein, Tb927.8.5240, and its role in determining sensitivity to human serum are unknown. The lysosomal membrane protein p67 is also shown.

To improve our understanding of sensitivity to human serum in African trypanosomes, we focussed on the role of the cysteine peptidase inhibitor, ICP. Our chemical and genetic evidence are entirely consistent, and reveal CATL as the cathepsin primarily responsible for the decreased sensitivity to human serum seen following *ICP* deletion. Specifically, chemical inhibition or knockdown of the individual cathepsins in an *icp* null background revealed that only CATL can resist human serum; CATB depletion had no detectable effect on this phenotype. CATL has been shown to accumulate in the lysosome [30], and is responsible for proteolysis of the transferrin receptor [36] and of anti-parasite IgG [28]. The lysosome is also the major site of action of APOL1 [13], the lytic component of both TLF1 and TLF2. Thus, CATL may target TLF, and possibly APOL1, for destruction in the lysosome (Figure 8). ICP, therefore, naturally maintains sensitivity to human serum, possibly by restricting the proteolytic degradation of TLF (or APOL1) in the lysosome. This is consistent with previous pulse chase experiments that found little proteolytic degradation of the lytic factor and its components in *T. b. brucei* [37], thereby allowing APOL1 to form membrane-spanning pores, leading to lysosomal swelling and cell lysis. Unmasking of the CATL activity only in the absence of ICP confirms natural control of this cathepsin, as suspected, by ICP.

Using a similar RNAi-screening approach, uptake of the anti-trypanosomal drug, suramin, was shown to be via receptor-mediated endocytosis in *T. b. brucei* [25]. The identification of only p67 by both screens suggests distinct uptake and trafficking factors and mechanisms involved in suramin uptake following association with the type-I *trans*-membrane glycoprotein, ISG75 [25], and TLF-uptake following association with the GPI-anchored HpHbR [11]. Interestingly, CATL has now been linked to both suramin efficacy [25] and human serum toxicity (this study) but, while CATL can protect *T. b. brucei* from killing by human serum, it sensitises *T. b. brucei* to killing by suramin. This suggests that suramin, a napthylamine, is liberated in active form by lysosomal proteolysis, while we suggest that TLF (APOL1) is degraded by lysosomal proteolysis.

Trypanosomal cathepsins are targets of ongoing drug development [29], [38], [39]. Although it was previously suggested that CATB was an appropriate drug target [31], more recent genetic and chemical evidence indicates that CATL is the essential cysteine peptidase of *T. b. brucei* and the most appropriate target [25], [29]. Our current findings also support this view. In this context, it is worth considering the potential impact of therapy targeting the essential cysteine peptidase activity. Our results indicate little impact of exposure to such inhibitors on human serum sensitivity in *T. b. brucei*. However, cysteine peptidases may be more active in *T. b. gambiense* [19] and/or *T. b rhodesiense*, possibly due to selective pressure through TLF exposure, and CATL inhibition could act synergistically with TLF in this case, increasing sensitivity to lytic activity and presenting a novel rational approach to therapy. On the other hand, *T. b. gambiense* and *T. b. rhodesiense* rely upon lysosomal/endosomal VSG variants to resist the toxic effects of APOL1. Reduced proteolysis of these factors [19] could increase parasite resistance to human serum, meaning that targeting CATL could represent a risky therapeutic strategy. Indeed, FMK024 exposure leads to an accumulation of SRA in the lysosome of *T. b. brucei* engineered to express this VSG-variant [2]. It will clearly be important to develop an improved understanding of the interplay among these factors in human-infective trypanosomes.

We link four factors to human serum sensitivity using a genome-scale loss-of-function screen in *T. b. brucei*. These include all three expected factors, based on previous reports, and a novel putative *trans*-membrane channel. It is interesting to note that, in the case of ICP, we uncovered a gain-of-function phenotype using a loss-of-function screen; this is possible when one protein antagonises the action of another. Our findings indicate that CATL can resist lysis by human serum, and this has important implications, since CATL is a promising potential drug target. In addition, the novel link to a putative *trans*-membrane channel presents an excellent candidate that may facilitate TLF transit. Notably, the gene encoding TgsGP is not present in *T. b. gambiense* group 2 [40], indicating a distinct human serum resistance mechanism in these parasites, and the main route of entry for TLF2 in *T. b. brucei* is thought to be independent of HpHbR [11], [19]. The outputs from our screen and our studies on ICP and CATL shed light on mechanisms of toxin delivery and stability in African trypanosomes and should facilitate studies aimed at understanding the multiple mechanisms employed by *T. b. gambiense* and *T. b. rhodesiense* to resist lytic factors in humans and other primates.

## Materials and Methods

### *T. b. brucei* strains

MITat 1.2 clone 221a 2T1 bloodstream-form *T. b. brucei* were maintained and manipulated as previously described [33]. Transformants were selected in blasticidin (10 µg/ml), hygromycin (2.5 µg/ml) or G418 (2 µg/ml), as appropriate. For growth assays, cells were seeded at ∼10^5^/ml, counted using a haemocytometer, and diluted back every 24 hours, as necessary, for up to seven days in the absence of antibiotics.

### NHS sensitivity analysis

To determine NHS EC_50_, cells were seeded at 2×10^3^ ml^−1^ in 96-well plates in a 2-fold dilution series of NHS (pooled mixed gender; Sera Laboratories International), starting from 0.01%; assays were carried out in the absence of antibiotics. After ∼3 days growth, 20 µl of 125 µg/ml resazurin (Sigma) in PBS was added to each well and the plates incubated for a further 6 hours at 37°C. Fluorescence was determined using a fluorescence plate reader (Molecular Devices) at an excitation wavelength of 530 nm, an emission wavelength of 585 nm and a filter cut-off of 570 nm [41]. To analyse the combined effect of NHS and FMK024 treatment, isobologram analysis was carried out using a checkerboard approach, as previously described [42]. Data were processed in Excel, and non-linear regression analysis carried out in GraphPad Prism.

### Selective screening of a genome scale *T. b. brucei* RNAi library

The bloodstream-form *T. b. brucei* RNAi library [24] was thawed into 100 ml HMI-11 media (Life Technologies) containing 10% foetal bovine serum (FBS; Sigma) at a density of approximately 1×10^5^/ml. RNAi was induced in 1 µg/ml tetracycline for 24 hours prior to the addition of 0.0005% NHS; RNAi induction and NHS selection were maintained throughout. Daily counts were carried out using a haemocytometer, and the total population was maintained at no lower than 20 million cells for the duration of selection. Once robust growth had been achieved, the inducibility of the selected phenotype was tested [25] (Figure 1D) and genomic DNA prepared for RNAi target identification.

### Sequencing and mapping

The RNAi cassettes remaining in the NHS-selected library were specifically amplified from genomic DNA using the LIB2f/LIB2r primers [43] producing a ladder of bands ranging in size from 0.25–1.5-kbp following agarose gel-electrophoresis (data not shown). High-throughput sequencing of the amplified DNA was carried out on an Illumina platform (Beijing Genome Institute). Using paired 150-bp sequencing reads; presence or absence of a 14-bp RNAi-construct signature was recorded in the FASTQ header line. Sequence reads were then trimmed to remove lower-quality sequences and mapped to the *T. b. brucei* reference genome (release 4.2) using *bowtie* [44]. BAM files were processed using the SAMtools bioinformatics suite [45]. The maps were explored visually in Artemis, and plots were derived using the Artemis graph tool and processed in Adobe Photoshop Elements 8.0. Stacks of reads that included the 14-bp signature on the positive strand were used to define RNAi target fragment junctions and to assign high-confidence hits as those identified by >1 RNAi target fragment.

### Plasmid and strain construction

*ICP* deletion was carried out as described [28], except that targeting fragments were cloned in pBSD, and the blasticidin-S-deaminase cassette was then replaced with a neomycin phosphotransferase cassette to generate pBSDΔICP and pNPTΔICP constructs. We *C*-terminally cMyc-tagged an endogenous copy of CATB at a native allele [46]. A 990 bp CATB *C*-terminal fragment minus the stop codon was cloned into pNATx^12MYC^ [46]; the construct was linearised with *Pst*I prior to transfection. Stem-loop RNAi constructs targeting CATB (Tb927.6.560), CATL (Tb927.6.960-1060) or Tb927.8.5240 were generated in pRPa^iSL^ [46]; 206 bp (CATB), 578 bp (CATL) and 417 bp (Tb927.8.5240) target fragments were designed using the RNAit primer design algorithm to minimise off-target effects [47]. pRPa^iSL^ constructs were linearised with *Asc*I to enable targeted integration at the rDNA spacer ‘landing pad’ locus in 2T1 bloodstream form *T. b. brucei* [33]. Details of all oligonucleotides are available on request. Linearised constructs were transferred to *icp* null or wild-type 2T1 *T. b. brucei* using a nucleofector apparatus (Lonza) in conjunction with cytomix or T-cell nucleofection solutions. Protein expression following RNAi depletion was analysed by SDS-PAGE and western blotting with anti-CATL and anti-cMyc, using standard protocols [48].

### cDNA synthesis and qPCR analysis following Tb927.8.5240 RNAi

For each cell line and treatment, 2 µg RNA was DNase-treated and reverse-transcribed using the Superscript VILO cDNA synthesis kit (Invitrogen). 100 ng (RNA-equivalent) cDNA was subjected to qPCR using the Quantitect SYBR Green PCR kit (Qiagen) and primer pairs specific for telomerase reverse transcriptase (*TERT*; Tb927.11.10190) and Tb927.8.5240 (details of primer sequences are available on request). *TERT* was used as a reference for normalisation of gene expression, as previously described [49]. qPCR reactions were carried out in a Rotor-gene 3000 (Corbett Research), using the following cycling conditions: 95°C (15 minutes), followed by 40 cycles of 94°C (15 seconds), 58°C (30 seconds), and 72°C (30 seconds). Standard curves, derived from a series of 10-fold dilutions of the target PCR products, were used to determine reaction efficiency. Fold-change in gene expression was calculated by the ΔΔCt method [50].

## Supporting Information

Text S1 **Analysis of Tb927.8.5240 knockdown by reverse-transcriptase quantitative PCR.** Mean C_t_ data were used to calculate the ΔΔC_t_ values and fold-change in gene expression of Tb927.8.5240 following RNAi induction, relative to the normalisation gene (*TERT*).
(DOCX)
