# Supplementary material for: Cathepsin-L Can Resist Lysis by Human Serum in Trypanosoma brucei brucei
Source: PLoS Pathog. 2014 May 15;10(5):e1004130. doi: 10.1371/journal.ppat.1004130 (PMC4022737; doi:10.1371/journal.ppat.1004130)
Supplement: Text S1 — Analysis of Tb927.8.5240 knockdown by reverse-transcriptase quantitative PCR. Mean Ct data were used to calculate the ΔΔCt values and fold-change in gene expression of Tb927.8.5240 following RNAi induction, relative to the normalisation gene (TERT). (DOCX) [file ppat.1004130.s001.docx]

C_t_ data from reverse transcriptase qPCR of Tb927.8.5240 RNAi cell lines.

|  |  |  | ***Tb927.8.5240*** | | | ***TERT* (*Tb927.11.10190*)** | | |
| --- | --- | --- | --- | --- | --- | --- | --- | --- |
| **Clone** | **Condition** | **Replicate** | **C_t_** | **Mean C_t_** | **SD** | **C_t_** | **Mean C_t_** | **SD** |
| 1 | -tet | a | 15.31 | 15.27 | 0.17 | 16.61 | 16.73 | 0.46 |
|  |  | b | 15.25 |  |  | 16.10 |  |  |
|  |  | c | 15.34 |  |  | 17.07 |  |  |
|  |  | d | 15.17 |  |  | 17.43 |  |  |
|  |  | e | 15.53 |  |  | 16.70 |  |  |
|  |  | f | 15.03 |  |  | 16.50 |  |  |
|  | +tet (72 hours) | a | 17.05 | 16.96 | 0.15 | 16.49 | 16.65 | 0.31 |
|  |  | b | 16.69 |  |  | 16.82 |  |  |
|  |  | c | 17.09 |  |  | 16.51 |  |  |
|  |  | d | 16.96 |  |  | 16.95 |  |  |
|  |  | e | 17.04 |  |  | 16.94 |  |  |
|  |  | f | 16.91 |  |  | 16.16 |  |  |
| 2 | -tet | a | 15.22 | 15.47 | 0.25 | 17.18 | 17.14 | 0.25 |
|  |  | b | 15.31 |  |  | 17.13 |  |  |
|  |  | c | 15.72 |  |  | 16.98 |  |  |
|  |  | d | 15.53 |  |  | 17.14 |  |  |
|  |  | e | 15.24 |  |  | 16.83 |  |  |
|  |  | f | 15.80 |  |  | 17.58 |  |  |
|  | +tet (72 hours) | a | 17.03 | 16.89 | 0.36 | 16.95 | 16.47 | 0.26 |
|  |  | b | 17.47 |  |  | 16.46 |  |  |
|  |  | c | 16.83 |  |  | 16.45 |  |  |
|  |  | d | 16.99 |  |  | 16.22 |  |  |
|  |  | e | 16.62 |  |  | 16.49 |  |  |
|  |  | f | 16.42 |  |  | 16.24 |  |  |
| 3 | -tet | a | 16.04 | 15.76 | 0.27 | 16.97 | 16.92 | 0.14 |
|  |  | c | 15.66 |  |  | 16.74 |  |  |
|  |  | b | 15.36 |  |  | 17.06 |  |  |
|  |  | d | 15.88 |  |  | 17.09 |  |  |
|  |  | e | 15.58 |  |  | 16.87 |  |  |
|  |  | f | 16.03 |  |  | 16.82 |  |  |
|  | +tet (72 hours) | a | 17.34 | 17.36 | 0.20 | 16.39 | 16.62 | 0.15 |
|  |  | b | 17.26 |  |  | 16.53 |  |  |
|  |  | c | 17.16 |  |  | 16.73 |  |  |
|  |  | d | 17.65 |  |  | 16.56 |  |  |
|  |  | e | 17.21 |  |  | 16.71 |  |  |
|  |  | f | 17.54 |  |  | 16.80 |  |  |

ΔΔC_t_ data from reverse transcriptase qPCR analysis of the Tb 927.8.5240 RNAi cell lines. Mean C_t_ values in Supplementary Table 1 were used to calculate fold-change in gene expression of Tb927.8.5240 in the RNAi cell lines, relative to the normalisation gene (*TERT*).

| **Clone** | **ΔC_t_ induced** | **ΔC_t_ uninduced** | **ΔΔC_t_ (induced-uninduced)** | **Fold change** |
| --- | --- | --- | --- | --- |
| 1 | 0.31 | -1.46 | 1.77 | 0.29 |
| 2 | 0.42 | -1.67 | 2.09 | 0.23 |
| 3 | 0.74 | -1.16 | 1.90 | 0.27 |
|  |  |  |  |  |
|  |  |  | Mean | 0.26 |
|  |  |  | SD | 0.03 |
